# Sweet Bones: The Pathogenesis of Bone Alteration in Diabetes

**DOI:** 10.1155/2016/6969040

**Published:** 2016-09-29

**Authors:** Mohammed Al-Hariri

**Affiliations:** Department of Physiology, College of Medicine, University of Dammam, P. O. Box 2114-31451, Dammam, Saudi Arabia

## Abstract

Diabetic patients have increased fracture risk. The pathogenesis underlying the status of bone alterations in diabetes mellitus is not completely understood but is multifactorial. The major deficits appear to be related to a deficit in mineralized surface area, a decrement in the rate of mineral apposition, deceased osteoid surface, depressed osteoblast activity, and decreased numbers of osteoclasts due to abnormal insulin signaling pathway. Other prominent features of diabetes mellitus are an increased urinary excretion of calcium and magnesium, accumulation of advanced glycation end products, and oxidative stress leading to sweet bones (altered bone's strength, metabolism, and structure). Every diabetic patient should be assessed for risk factors for fractures and osteoporosis. The pathogenesis of the bone alterations in diabetes mellitus as well as their molecular mechanisms needs further study.

## 1. Introduction

Diabetes mellitus is a common chronic hyperglycemic, prevalent disease, with significant associated mortality and morbidity that affects millions of population worldwide. It is associated with a variety of complications that are well known to healthcare providers. In time, the bones may also be affected, in addition to many other organs. However, the status of bones as well as their disorders in patients with diabetes mellitus has received very little attention. This is surprising because bone disease in diabetes mellitus is probably as old as the disease itself since descriptions of bone disease in diabetes can be traced as far back as the 1920s and is as old as insulin itself [1]. Vestergaard et al. (2009) concluded that diabetes, whether type 1 diabetes (T1D) or type 2 diabetes (T2D), seems to carry an increased risk of fractures [2]. Recent studies have identified an impairment of bone quality and a higher risk of fracture, in those with T2D [3].

During the last two decades, studies reported that T2D is associated with up to three times increased risk of fracture [4, 5].

## 2. Pathogenesis of Sweet Bones

**Figure 1:**
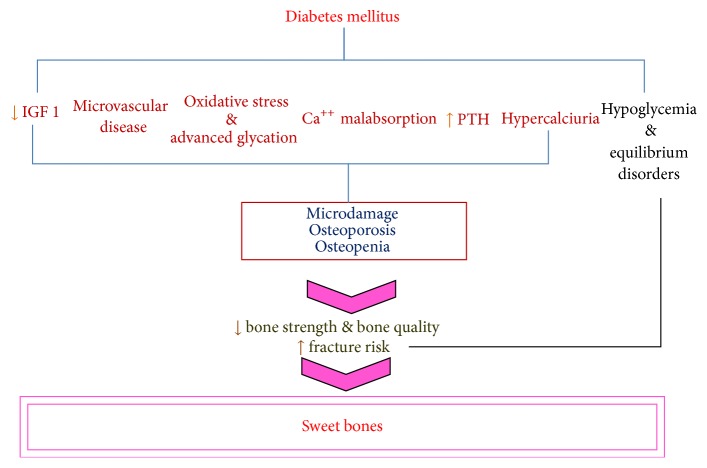
The pathogenesis of bone alteration in diabetes.

There are different mechanisms that can be proposed to explain the pathogenesis of sweet bones in diabetes mellitus, summarised in Figure 1.

Insulin signaling has a metabolic and mitogenic effect on osteoblast cells. Current evidence in experimental models with impaired insulin signaling exhibited both metabolic and bone phenotypes, including obesity, insulin intolerance/resistance, and symptoms of low bone mass [6] and the qualitatively different effects of T1D and T2D on bone mass are consistent with the opposing insulin-secretory states [hypoinsulinaemia versus hyperinsulinaemia] [7].

Diabetes could impact bone through several mechanisms, some of which may have contradictory effects. There are many reasons why diabetics are likely to develop bone disease and sustain fractures [8]. Many studies have suggested that low bone mineral density (BMD) is already apparent at the time of diagnosis [9, 10].

In T1D, for instance, patients may not attain the full potential of peak adult bone mass because of lower insulin-like growth factor 1 levels and the catabolic effects of frequent uncontrolled hyperglycemia during critical growth period [11]. Altered vitamin D and calcium metabolism due to hyperglycemia in diabetes mellitus can lead to low bone mass and increase chances of fractures [12, 13].

A number of studies demonstrated that osteopenia and osteoporosis are frequent complications of T1D [14], as a result of increased oxidative stress [15], as well as to the alteration of osteoblastic function [16].

Since almost 40% of the skeletal calcium is accumulated between the ages of 10 and 15 years, precisely the time when diabetes control may not be optimal, developing diabetes at this critical juncture may adversely affect the potential to achieve peak adult bone mass. It was reported that a significant number of patients with diabetes may have associated hypovitaminosis-D and calcium malabsorption or both [17, 18], low body mass, overt or subclinical malabsorption such as cystic fibrosis or celiac sprue [19, 20], and a higher incidence of subclinical eating behavior disorders, contributing to poor weight maintenance and/or relative malnutrition [21].

With the increasing life expectancy of many diabetic patients, the age related decline in osteoblast function contributes to the pathogenesis of bone loss and recurrent fractures [22].

Diabetes mellitus is known to cause advanced glycation of a variety of proteins that may also include glycation of type I collagen in bone and thus compromise its integrity [11].

Furthermore, peripheral vascular disease is common in this disease which may also contribute to the bone disease and fractures. Reduced interstitial bone fluid flow as a result of diabetic microvascular disease may result in osteocyte apoptosis and reduced osteocyte density leading to increased fragility of bone [23].

In contrast, the occurrence of bone disease in T2D presents an apparent paradox [8], but Takeuchi (2009) in his review reported that bone fragility due to poor bone quality is a major problem in patients with T2D [24]. It has been documented that T2D cases with high bone turnover assuredly predisposed to osteoporosis [25]. Obesity prevalent in T2D is strongly associated with higher BMD probably through mechanical loading and hormonal factors including insulin, estrogen, and leptin [26, 27].

A long-term T1D model showed that diabetic bones display specific defects of bone mineralization, including decreased hydroxyapatite crystal perfection, decreased calcium-to-phosphate composition of the ash, and decreased ash content in certain bones such as the tibial metaphysis. It also found that the bones from diabetic animals exhibited reduced strength-related properties, along with a compensatory increase in stiffness, suggesting a possible alteration in bone crystal structure [28].

In a number of T1D animal studies, histomorphometric analyses have shown that, irrespective of the model used, insulin-deficient rats may exhibit reduced or absent bone formation and this decline is appreciated in relation to all bone surfaces examined [29, 30].

The major deficits in the insulin-deficient models appear to be related to a deficit in mineralized surface area, a decrement in the rate of mineral apposition, deceased osteoid surface, depressed osteoblast activity, and decreased numbers of osteoclasts [31], leading to an overall depression in remodeling of bone in the untreated insulin-deficient state.

Moreover, unlike patients with T1D, T2D patients have higher levels of insulin-like growth factor 1, which is known to stimulate bone formation. Indeed, there is suggestive evidence that age related bone loss is attenuated and bone turnover is either normal or reduced in patients with T2D ([32] and [33]). However, the existence of an elevated fracture risk in T2D, despite the underlying hyperinsulinaemia, suggests the involvement of other potential pathogenic influences (e.g., hyperglycemia, diabetic complications, and lifestyle factors) on bone [7]. In the experimental model, the skeletal fragility in T2D may arise from reduced transverse bone accrual and increased osteoclastogenesis during growth that is accelerated by the diabetic/hyperinsulinemic milieu [34]. Therefore, bone density at relevant measurement sites may not reflect the true quality of the skeleton in patients with T2D; in other words the quantity of bone may be normal but the quality is not.

All of these confounding variables may have independent negative impacts upon bone mineral acquisition in diabetes mellitus and, ultimately, on peak bone mass. On the other hand, low levels of insulin associated with T1D and the progression of T2D may cause reductions in BMD. Hyperglycemia generates a higher concentration of advanced glycation end products in collagen that may reduce bone strength [35].

T1D in humans has frequently been shown to be associated with reduced bone mass [36] and a reduced bone mineral content [37]. The percentage of patients reported to have osteopenia ranges from 18 to 54% [38]. Low turnover osteopenia with reduced mineral content has also been well documented in experimental models of T1D, such as streptozotocin-induced diabetes [39]. Another prominent feature of human and experimental T1D is an increased urinary excretion of calcium and magnesium [40].

Plasma calcium concentration in the rat is normally maintained in the face of such renal losses; a decreased bone mineral content may therefore represent an unfortunate consequence of the marked hypercalciuria serving to maintain normocalcaemia under these conditions [40].

Delayed and impaired fracture's healing in patients with diabetes mellitus has been described in many studies [41, 42]. Diabetes impairs the production of critical growth factors such as transforming growth factor-beta, insulin-like growth factor 1, vascular endothelial growth factor, and platelet-derived growth factor at the fracture site during the early phases of diabetic fracture healing which has been associated with decreased cell differentiation and proliferation [43, 44]. Previous studies have demonstrated a reduction in collagen synthesis in diabetic rats [45] which ultimately influences bone healing.

Some fractures that frequently occur in diabetic patients may not be related to the systemic effect of diabetes on the skeleton but rather may be due to hypoglycemia, neuropathy, loss of proprioception balance, and coordination that are common in this disease and an important risk factor for falling [46, 47].

## 3. Prevention of Sweet Bones

**Figure 2:**
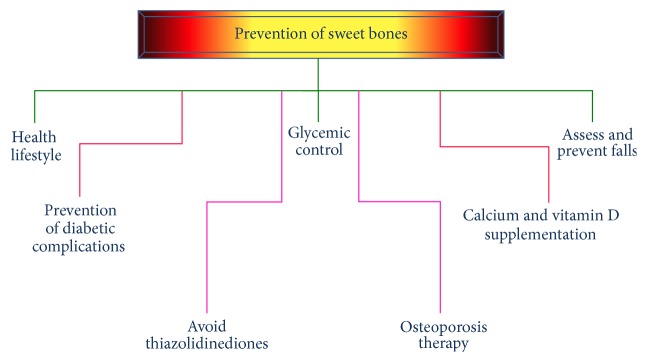
Preventive measures of sweet bones.

Prevention of any disease is a laudable goal. When this is applied to diabetes mellitus, it gains further importance because of the fact that this disease is gaining epidemic proportion and the cost of treatment of its complications. Sweet bone can be prevented or delayed by making some changes in lifestyle and weight loss, accompanied by increased physical activity to prevent bone loss. Intensive insulin therapy is the standard treatment for T1D and seems to be associated with improved skeletal health [48, 49]. Systematic screening for diabetic complications such as polyneuropathy, retinopathy, and nephropathy is important. Laser therapy might prevent progression of advanced retinopathy and help to maintain vision [50]. Deficiencies of calcium and vitamin D in patients with diabetes mellitus should be treated. Vitamin D supplementation should ensure a serum 25-hydroxyvitamin D level of 75 nmol/L [51]. Attention should be paid to the use of thiazolidinediones, especially in postmenopausal women with T2D. It causes bone loss accompanied by decreased osteoblast activity and bone formation [52]. Assessment of osteoporosis is similar in patients with and without diabetes mellitus. Selection of specific osteoporosis drugs is frequently based on comorbidities [53] (Figure 2).

## 4. Conclusion

Nevertheless, awareness of sweet bone in a patient with diabetes mellitus is important in clinical practice. Early recognition and appropriate intervention are essential in avoiding sweet bones and its consequences in diabetic patient.

Every diabetic patient should be assessed for risk factors for fractures and osteoporosis according to the guidelines established by The International Society for Clinical Densitometry and The National Osteoporosis Foundation. The pathogenesis of the bone alterations in diabetes mellitus as well as their molecular mechanisms needs further study.
